# An Overview of Neovaginal Reconstruction Options in Male to Female Transsexuals

**DOI:** 10.1155/2014/638919

**Published:** 2014-05-26

**Authors:** Marta Bizic, Vladimir Kojovic, Dragana Duisin, Dusan Stanojevic, Svetlana Vujovic, Aleksandar Milosevic, Gradimir Korac, Miroslav L. Djordjevic

**Affiliations:** ^1^University Children's Hospital, Tirsova 10, 11000 Belgrade, Serbia; ^2^Institute for Child and Mother's Health “Dr Vukan Cupic”, Radoja Dakica 6-8, 11070 Belgrade, Serbia; ^3^Clinic of Psychiatry, Clinical Centre of Serbia, Pasterova 2, 11000 Belgrade, Serbia; ^4^School of Medicine, University of Belgrade, Dr Subotica 8, 11000 Belgrade, Serbia; ^5^Clinic for Gynecology and Obstetrics “Narodni Front”, Kraljice Natalije 62, 11000 Belgrade, Serbia; ^6^Institute of Endocrinology, Diabetes and Metabolic Diseases, Clinical Centre of Serbia, Dr Subotica 13, 11000 Belgrade, Serbia; ^7^Clinical Hospital Centre “Zvezdara”, Dimitrija Tucovica 161, 11050 Belgrade, Serbia; ^8^Faculty of Dental Medicine, University of Belgrade, Rankeova 4, 11000 Belgrade, Serbia

## Abstract

Transsexualism is a complex condition in which the person experiences the inconsistency between the desired gender and their biological gender. Absence of the vagina is devastating in male to female transsexuals. Creation of the neovagina is the main surgical problem in these patients. Historically, beginnings of the neovaginal creation have their roots in the treatment of Mayer-Rokitansky syndrome and conditions such as cloacal anomalies, certain intersex disorders, vaginal malignancies, or severe vaginal trauma, but have more recently found great purpose in male to female sex reassignment surgery. Many operative procedures have been described but none is ideal. Therefore, the search for new, improved solutions continues. In neovaginoplasty reconstruction of the vulvovaginal complex is performed in its entity. The gold standard in neovaginal reconstruction in male to female sex reassignment surgery is penile skin inversion technique with or without scrotal flaps, which enables adequate sensation of the neovagina, good neovaginal depth, good erotic sensitivity of the neclitoris, and esthetically acceptable labia minora and maiora.

## 1. Introduction

Transsexualism, as a condition, is widely misunderstood. In the light of their gender identity issues, transsexuals are generally perceived as emotionally unstable persons, incapable of coping with their everyday lives. The term “transsexual” came into professional and public usage in the 1950's to denote a person who aspired to or actually lived in the anatomically opposite gender, regardless of whether they had undergone a gender reassignment surgery and/or were undergoing hormonal treatment. Recently, however, the general public has become somewhat more aware and more accepting of transsexuals. In the International Classification of Diseases (ICD-10)* “transsexualism”* is described as a desire to live and be accepted as a member of the opposite sex, usually accompanied by a sense of discomfort with, or inappropriateness of, one's anatomic sex, and a wish to undergo surgery and hormonal treatment to make one's body as congruent as possible with one's preferred sex [1]. There are different studies regarding the prevalence of transsexualism in general population accounting for 1 : 7400 to 1 : 42000 in assigned males and 1 : 30040 to 1 : 104000 in assigned females [2–8]. Sex reassignment surgery (SRS) is the last step in an individual's transition to the preferred gender. It comprises surgical procedures that will reshape the individual's body into a body with the appearance of the desired gender. In male to female transsexual patients, the surgeon needs to reconstruct female genitalia, as well as to remodel a male body into a female-looking body.

One of the first surgeons, who successfully performed gender reassignment surgery from female to male, and later from male to female, was a plastic surgeon Sir Harold Gillies. Together with Dr. Ralph Millard, he performed a vaginoplasty using the skin flap technique that became the standard for the next few decades [9].

In male to female genital reassignment surgery, the objective is to create a vagina and external genital organs that are as feminine as possible in appearance, with no scars or traumatic postoperative neuromas, as concluded by Karim et al. [10]. The urethra is shortened, so that the urinary stream points downwards in the sitting position. In this procedure, it is important to avoid formation of fistulas or strictures. An ideal neovagina should be moist, elastic and hairless, no less than 10 cm in depth and about 3-4 cm in diameter, with no introital stenoses. Its innervation should provide adequate sensation, to achieve a satisfactory level of erogenous stimulation during sexual intercourse [10]. Clitoris should be small and obscured, but sensitive, offering the patient complete arousal. Labia minora and majora should be as similar as the female vulva as possible and not bulky.

A variety of surgical options exists for vaginal reconstruction. In this article, we review several different reconstructive approaches. Regardless of reconstruction method, the goals remain the same: creating a functional and aesthetically acceptable vagina and vulva, with a normal voiding function and satisfactory sexual function.

Nowadays, the two most widespread techniques for neovaginal reconstruction are “penile inversion technique”, with or without combining scrotal flaps and the use of intestinal pedicled transplants. Due to their importance, these two techniques will be discussed in more detail, compared to other reviewed techniques.

## 2. Surgical Techniques for Vaginoplasty

### 2.1. Nongenital Skin Grafts

One of the first techniques in neovaginal reconstruction in transsexual patients, introduced by Abraham, dates from nineteen thirties and comprises the use of skin grafts [11]. In his technique, the skin graft was draped, inside-out, over a sponge placed between the rectum and urethra to serve as a mold, in line with the technique first published by Abbe in congenital vaginal absence in nineteenth century [12], and popularized later in 1938 by Banister and McIndoe [13]. Split-thickness skin grafting is commonly associated with low morbidity. Because of its relative simplicity, this method gained worldwide acceptance. The advantage of nongenital skin graft technique is that it is a single stage procedure, yielding a hairless neovagina of sufficient depth and width, with low risk of postoperative complications. The disadvantages include neovaginal prolapse, scarring of the donor area, circular scarring present at the neovaginal introitus, the tendency of the skin graft to shrink, condylomatosis, intraepithelial neoplasia in combination with Human Papilloma Virus (HPV), carcinoma, poor erogenous sensation and absence of natural lubrication [14–16].

### 2.2. Penile Skin Graft

Fogh-Anderson was the first to report the creation of a neovagina from a full thickness skin graft harvested from penile skin, as a male-to-female gender reassignment surgery in transsexuals, in 1956 [14, 17]. The skin graft was fixed to a mold, in line with the “McIndoe technique.” This technique is also a single stage procedure; its other advantage is that it uses hairless penile skin for the creation of a neovagina, with almost no visible scars to the donor area. In addition, the risk of postoperative complications is low, due to the fact that full-thickness skin grafts are less prone to contraction than split-thickness skin grafts. Still, intermittent dilatation is required in these patients as well, for several months postoperatively, to prevent shrinking of the neovagina. In addition, they may develop the same postoperative complications as the patients with split-thickness neovaginas, such as condylomatosis, intraepithelial neoplasia in combination with HPV, carcinoma, poor erogenous sensation and absence of natural lubrication [14–16].

### 2.3. Genital Skin Flaps

As stated previously, Gilles and Millard reported the use of penile skin flap in the creation of a neovagina in male to female transsexuals in 1957 [9]. This procedure requires several sub-procedures to form the new vagina. Following the usual bilateral orchiectomy, the penis is dissected into its anatomical components, that is, the corpora cavernosa, the glans cap with the urethra and the neurovascular bundle, and the vascularized penile skin [9, 18, 19]. Quite a few modifications of this technique have been described since, and it remains the gold standard in male to female sex reassignment surgery. There are generally three groups of such modifications: (a) Use of inverted penile skin on an abdominal pedicle, as the sole graft, in the form of an inside-out skin tube [9, 14]; (b) Splitting the pedicled penile skin flap to create a rectangular flap, which is then augmented by a rectangular scrotal skin flap with a posterior pedicle to increase the size of the neovagina [14, 20]; (c) The pedicled penile skin flap that can also be enlarged with a long vascularized urethral flap, which is harvested and then embedded in the penile skin-tube flap [21, 22]. The advantages of the penile skin flap technique in comparison to the skin grafts techniques include a decreased tendency to contract, sensation provided by the pedicle, absence of hair on the flap and a far less common occurrence of neovaginal prolapse. The disadvantages include postoperative use of vaginal dilatators for at least 6 months after the surgery, according to instructions. Limited vaginal depth due to the limitation of penile skin length and mobilization of the pedicle results in wider anterior commissure that leaves clitoris more exposed and more sensitive and sometimes even painful during recovery. In cases where the penile skin length is insufficient, the scrotal skin flap can be used; however, unless this region is subjected to laser hair removal pre-operatively, this will result in a partly hairy neovagina [23, 24].

**Figure 1:**
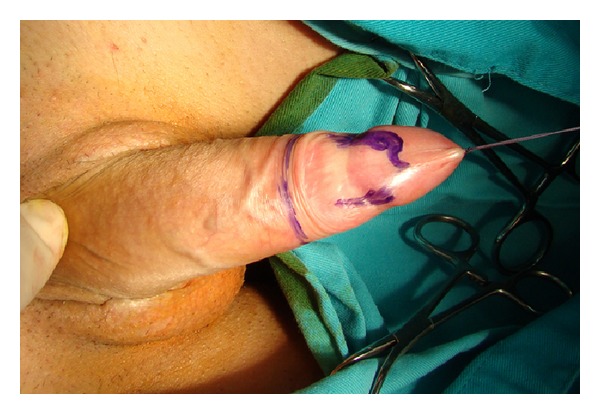
Marked incision lines for clitoroplasty and vaginoplasty.

**Figure 2:**
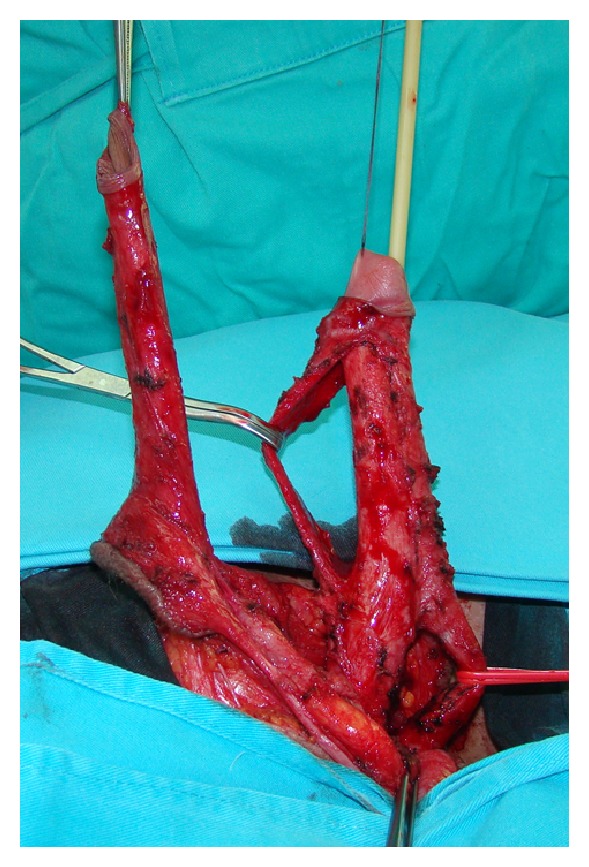
Freed penile skin, dissected neurovascular bundle and mobilized urethra.

**Figure 3:**
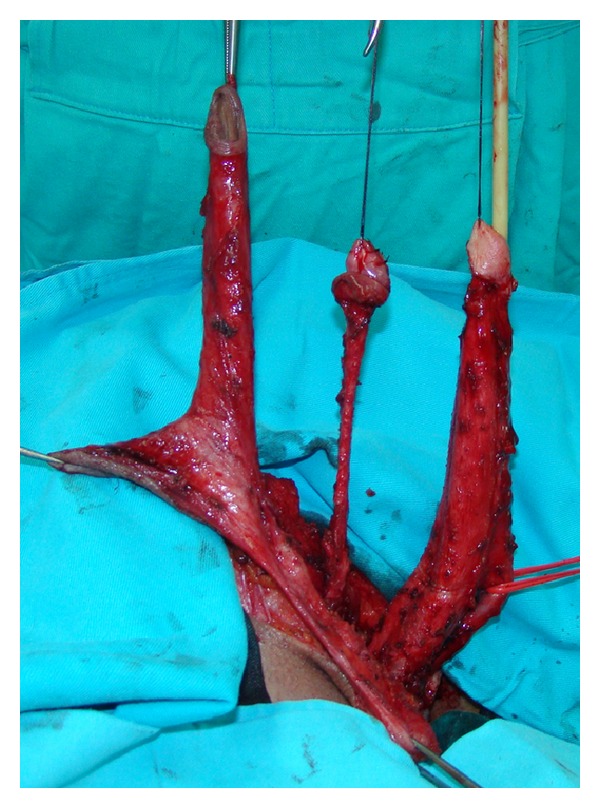
Penile disassembly is done. Conically shaped clitoris with preserved neurovascular bundle is created.

**Figure 4:**
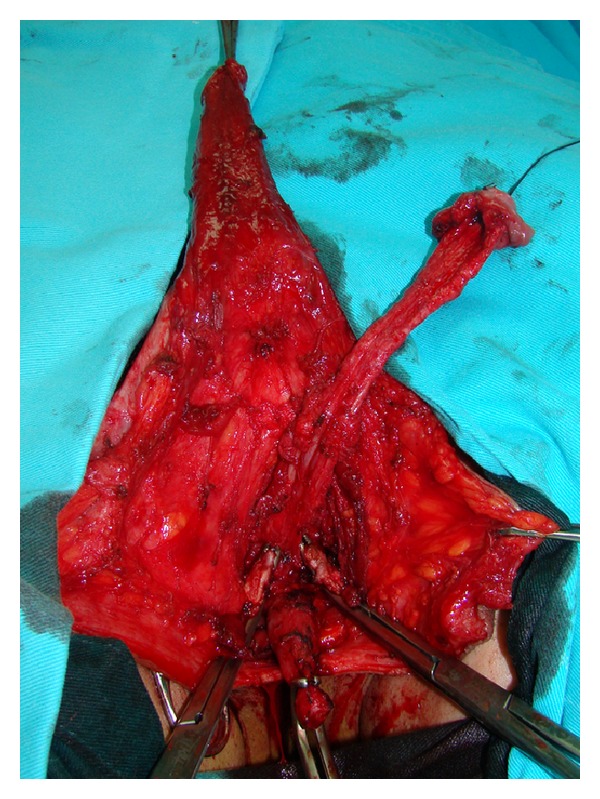
Removal of the corpora cavernosa deeply to their attachments on the pubic bones.

**Figure 5:**
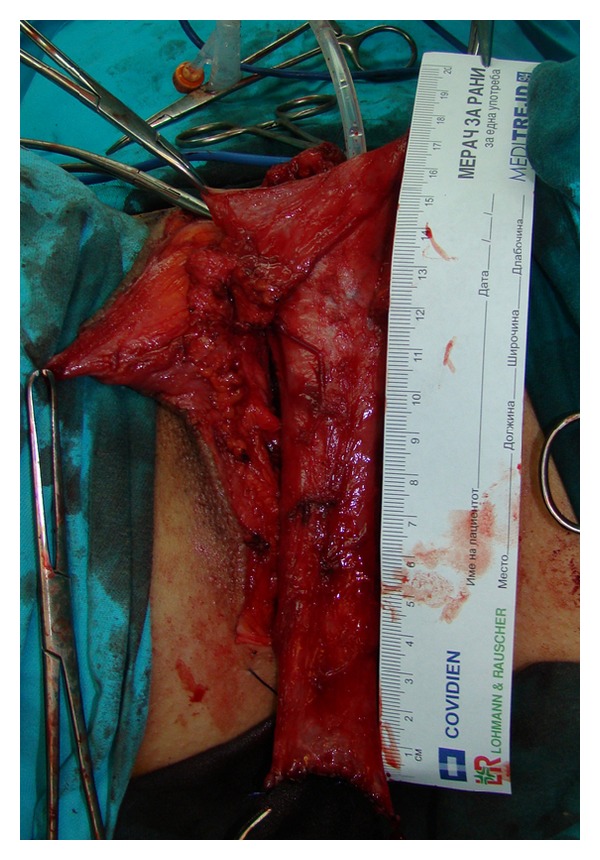
Long tube consisting of vascularized penile skin and urethral flap is inverted to form neovagina.

**Figure 6:**
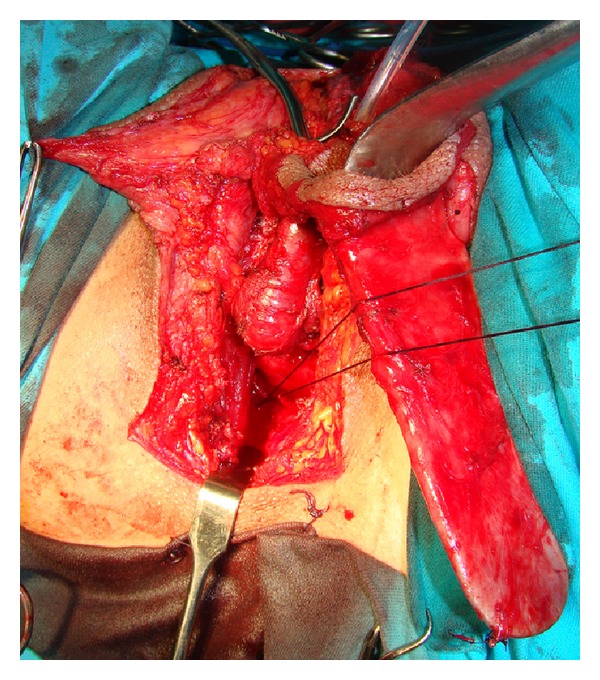
Neovagina is tied deeply to the sacrospinous ligament using Deschamps ligature carrier to prevent its prolapse.

**Figure 7:**
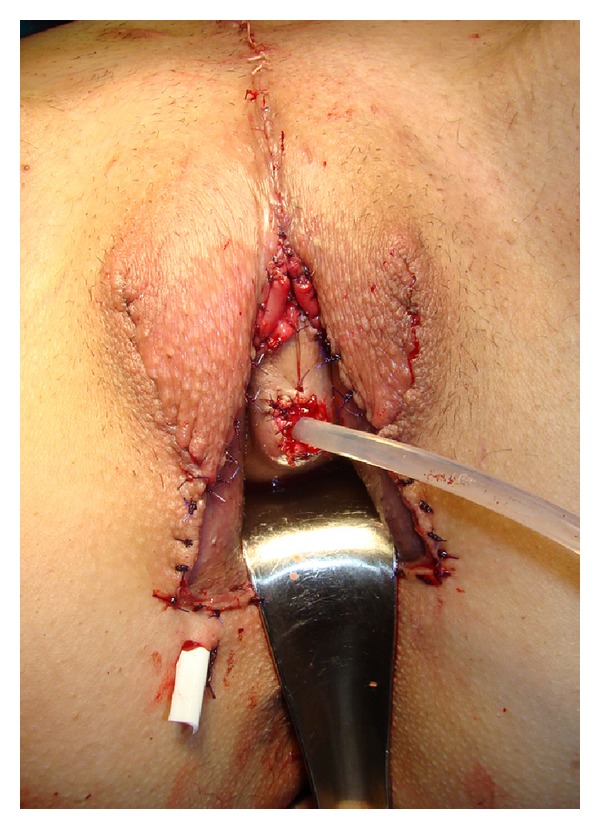
Outcome at the end of surgery.

One of the main issues is the corpora cavernosa dissection up to their attachments to the inferior ramus of the pubic bones [25]. In cases with very long corporeal crura, corporeal bodies are removed while the remnants of the corpora cavernosa (erectile tissue) are destroyed and the tunica albuginea sutured with absorbable sutures. This prevents any postoperative erection that can hinder future sexual intercourse or narrow the neovaginal introitus during arousal. To construct the new vagina, the skin of the penile body and prepuce (in uncircumcised patients) is harvested and then shaped into a vascularized island tube flap. Obtaining a long vascularized pedicle for the tube is of key importance, so the incision is made <2 cm above the base of the mobilized penile skin. At this point, the loose subcutaneous tissue allows for the formation of a long vascularized pedicle. At the base of the pedicle, a small incision is made to transpose the urethral flap [26]. The skin on the dorsal side of the tube flap is incised, leaving the vascularized subcutaneous tissue intact. Once a neovagina is created, a blunt dissection of the vaginal cavity is performed anterior to Denonvilliers fascia, taking care not to injure the rectum. A long-handled Deschamps ligature carrier preloaded with 2-0 absorbable suture is used to pierce the sacrospinous ligament medially to the ischial spine. The surgeon must pay attention not to place the suture close to the ischial spine to prevent injury of the pudendal nerve and internal pudendal vessels. The two ends of the suture are brought out so that the fixation stitches can be tied in place; one is passed through the skin and the other is passed through the urethral flap in the part situated in the distal third of the neovagina, after which the stitches are tied. In this way, a vaginopexy to the sacrospinous ligament is performed, with deep placement of the neovagina in the perineal cavity. This provides a good placement of the neovagina, avoiding its prolapse [22, 27, 28] (Figures 1, 2, 3,4,5, 6 and 7).

### 2.4. Nongenital Skin Flaps

As the male to female genital reconstructive surgery evolved, the search for a surgical technique with a more acceptable result for both patient and the surgeon lead to the use of nongenital skin flaps. There were different approaches from different centers, such as Cairins and De Villiers, who used medial thigh flap for neovaginal reconstruction in transsexual patients, and Huang who used inguinopudendal flaps to create the neovagina [29, 30]. Advantages of these techniques include the creation of a neovagina of an adequate depth, less risk of contraction and reduced period of postoperative dilatation compared to the nongenital skin graft technique. Disadvantages include scarring of the donor area, technically very demanding procedures and bulkiness of the flaps, with reduction of the neovaginal vault and no self-lubrication. Nevertheless, Karim et al. believe that nongenital skin flaps should be considered only as alternative surgical methods in failed primary vaginoplasties, where a pedicled intestinal transplant is still their method of choice [10, 14].

### 2.5. Pedicled Intestinal Transplant Vaginoplasty

**Figure 8:**
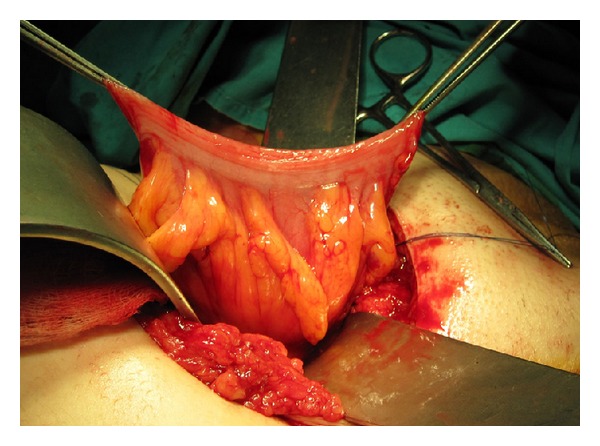
Harvested segment of sigmoid colon with its mesentery.

**Figure 9:**
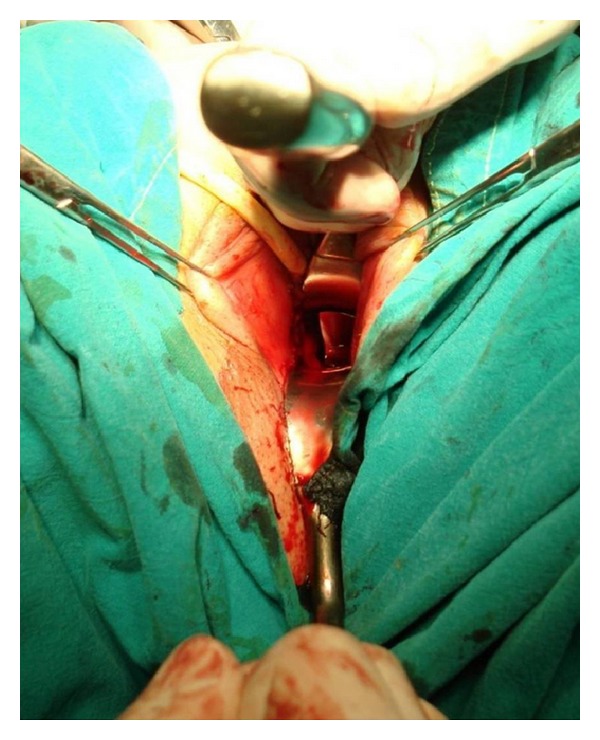
Anastomosis of the sigmoid colon with genital skin flaps, deeply hidden.

**Figure 10:**
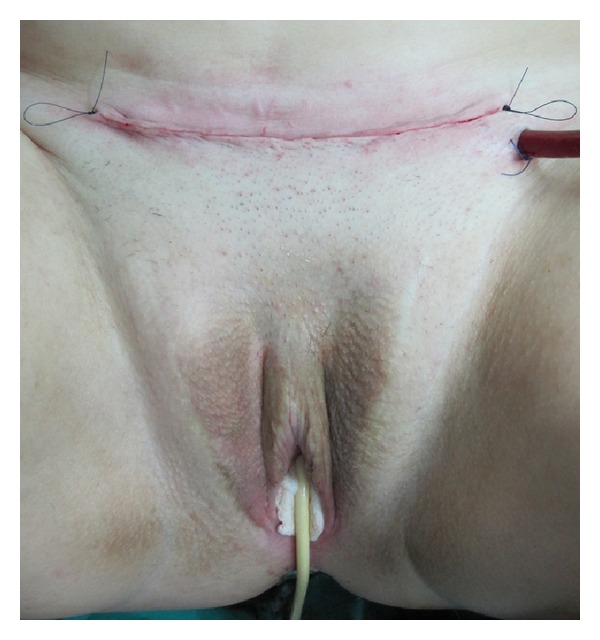
Appearance at the end of surgery.

Bowel vaginoplasty was first described by Sneguireff in 1892 using the rectum in the treatment of vaginal agenesis [31]. Later, in 1904, Baldwin reported the use of ileal segment in the treatment of congenital vaginal absence, but also suggested that the sigmoid colon might be used for the same purpose [32]. In male to female transsexuals, first mention of intestinal vaginoplasty dates from 1974, when Markland and Hastings used cecum and sigmoid transplants [14, 33]. Ileum is frequently used by many surgeons, but its mucosa produces more abundant and less lubricating secretions then the sigmoid segment [34]. Rectosigmoid vaginoplasty results in a well-proportioned, self-lubricating neovagina, which does not require postoperative dilatation for extended periods of time [35, 36]. Use of rectosigmoid colon as a pedicled flap for the creation of a neovagina is effective, being that a graft of sufficient length may be obtained, with an excellent blood supply that could prevent complications such as shrinkage or narrowing. This segment is thick-walled, large in diameter and can tolerate trauma better than small bowel, bladder or skin grafts, and subsequently leads to a decreased risk of bleeding after sexual intercourse. Introital or perineal skin flaps are designed in a manner that prevents purse string scarring; in addition, they are approximated to the sigmoid vagina, for the same reason. This is usually achieved via a “U” shaped incision posterior to the urethra [37]. Complete mobilization of the vascularized flaps is performed, in order to ensure that the introital opening is located as high as possible, which prevents mucosal prolapse, as well as to achieve a more pleasing esthetic result, as the anastamosis will be deeply set and thus obscured. (Figures 8, 9 and 10) Postoperative management is simple and easy. The production of mucus can lead to excessive discharge, which decreases dramatically after 3–6 months, regardless of the sigmoid segment length. Filipas et al. recommended a daily vaginal cleansing regiment to evacuate the mucus, for a period of one month [38]. The use of vaginal dilatators to maintain neovaginal patency is temporary and well tolerated by the majority of patients. Further disadvantages of intestinal vaginoplasty are the need for preoperative bowel preparation and additional abdominal surgery with intestinal anastomosis, which increases the risk of postoperative ileus. In addition, diversion colitis, as well as adenocarcinoma of neovagina, introital stenosis, mucocele and constipation have been reported [39–41], although with a low incidence.

Kim et al. recently concluded that rectosigmoid vaginoplasty is the best choice for male to female transsexuals who have previously undergone total penectomy and orchiectomy, or for those with previously failed skin vaginoplasty and for patients with Mayer-Rokitansky syndrome, as was also reported by Lima et al. [36, 42].

### 2.6. Clitoro-Labioplasty

Even though genital reconstructive surgery was primarily focused on finding the best technique of creating a neovagina that would allow the patient to engage in sexual intercourse, in recent years both patients and surgeons have become increasingly concerned with the aesthetic results of vulva and neoclitoris creation, as well as their ability to provide adequate erogenous sensation [43]. A section of penile skin base is used to form the labia minora, which are sutured to the de-epithelialized area of the neoclitoris; thus the neoclitoris is hooded with the labia minora. Excessive scrotal skin is removed and the remaining section used to form the labia majora. A small posterior base of the inverted Y-incision, advocated by Karim et al., facilitates the creation of an aesthetically appealing posterior commissure [44]. However, secondary corrections may be needed because of the changes in appearance, due to the healing process, usually one year post-operatively [45].

A very important surgical step is the creation of a well-vascularized neoclitoris during the primary vaginal reconstruction. The first report of the construction of a clitoris was published by Brown in 1976, using a reduced glans, attached to its dorsal penile neurovascular pedicle [46]. The high percentage of clitoral necrosis reported by Brown himself provoked other authors to seek new or modified techniques in clitoroplasty [47, 48]. Up to 1995, creation of the neoclitoris was not an important part of surgical standards in MTF sex reassignment surgery. Nowadays, the glans cap is divided into two parts, ventral and dorsal; the dorsal section of the glans is reduced by excising the central ventral tissue, leaving the sides of the glans intact. Lateral excisions on the glans are not recommended, to avoid injuring the neurovascular bundle, which enters the glans cap lateroventrally. However, the sides are de-epithelialized and sutured, to obtain a conical shape of the neoclitoris [49].

Additional incisions and corrections in pursuit of a perfect outcome can endanger the penile skin blood supply and survival of the neoclitoris and result in hypertrophic scar formation, wound dehiscence, or partial or complete necrosis of the flap due to the tension on suture lines. The widely accepted opinion is that it is better to wait with the corrective procedures until wound healing was complete, because no further operation will be necessary in most patients. In cases where the ventral space between the labia majora is too wide, simultaneous infrapubic double-Z-plasties can be performed with great success [24, 45].

The clitoris, its prepuce and labia minora remain among the most difficult structures to reconstruct. The ideal clitoro-labioplasty, which would yield a result resembling a biological female in every aspect, has not yet been achieved [14, 21].

## 3. Quality of Sexual Life

Most of the studies on transsexual patients focus on long-term psychological, surgical and physical health, while just a few focus on their sexual life after genital reassignment surgery [50, 51].

There are reports of sexual satisfaction after vaginoplasty in male to female patients, who were capable of achieving an orgasm in 70–80% [52]. As concluded by Lief and Hubschman, a patient can be sexually satisfied following a SRS, despite inadequate sexual functioning [53]. Our group also reported satisfactory results in 79% of male to female transgender patients following vaginoplasty involving penile skin combined with urethral flap [22, 26].

There are but a few reports presenting functional questionnaire-based results of vaginoplasty in patients after vaginal reconstruction, though majority of these studies are related to the patients with vaginal agenesis. Labus et al. reported absence of sexual dysfunction in 72.2% of their patients according the Female Sexual Function Index (FSFI), with depression symptoms in 22.2% [54]. Borkowski et al. assessed the functional results of Krzeski's cystovaginoplasty and patients' satisfaction using 18 parameters. Authors reported approximately 90% the overall satisfaction among the patients in the study group with the improvement of their sexual life and femininity [55]. Nevertheless, it is difficult to draw any general conclusion or to compare results between these studies, as different inventories and different surgical methods were used within different groups of patients.

Sexual expectations should be carefully discussed with the patients in preoperative preparation in order to help the patients deal with sexual changes and new function of new genitals. Aesthetic result, sexual arousal, lubrication as well as absence of pain during sexual intercourse are critical points of a successful male to female surgery. In their study, Weyers et al. reported that in average the transsexual women were very satisfied with their womanhood. Sexual functioning and sexual satisfaction assessed by FSFI was different in the study group according to the patient's sexual orientation, and markedly lower in transwomen with homosexual preference. As a generalized conclusion, authors stated that the modification of the FSFI questionnaire for the transwomen should be created to fully understand the sexual function and difficulties in transsexual women [56]. Psychological and psychosocial evaluation by structured interview and standardized questionnaires should be a part of this type of study. However, due to wide variations of circumstances and previous surgical involvement in these patients, such a study would still have certain limitations. In addition, it is difficult to identify a control group for any comparative [37, 54]. The lack of standardized methods for recording outcomes in terms of long term complications, as well as sexual function, limits the possibilities of direct comparison of results. Therefore, although a consensus on the ideal method of vaginal substitution may never be reached, efforts should be made to reach a consensus on the ideal way to follow these patients in the long term.

## 4. Conclusions

Functional satisfaction in patients undergoing male to female sex reassignment surgery is greatly reliant on adequate depth of the neovagina as well as on the sensation of the neoclitoris needed to achieve an orgasm. Overall satisfaction of the patients is also related to aesthetic appearance of the neocreated vulva, visibility of scars and need for repeat surgeries.

The goal of surgeons involved in male to female sex reassignment surgery should be to reach the procedure that will result in a neovagina that meets the patients' aesthetic and functional expectations.
